# Using a cardiac registry to highlight multidisciplinary care in pregnancy

**DOI:** 10.1007/s11845-026-04462-4

**Published:** 2026-06-05

**Authors:** Aoibhinn Smyth, C. O’Brien, M. Higgins, S. Corcoran, F. McAuliffe, C. Canniffe

**Affiliations:** 1 UCD Perinatal Research Centre, Obstetrics and Gynaecology National Maternity Hospital University College Dublin, Dublin, Ireland; 2 Midwifery, National Maternity Hospital, Dublin, Ireland; 3 Department of Obstetrics & Gynaecology, National Maternity Hospital, Dublin, Ireland; 4 Department of Cardiology, St Vincent’s University Hospital, University College Dublin, Dublin, Ireland

**Keywords:** Cardiac disease in pregnancy, Cardiac registry, Pregnancy heart team, Multidisciplinary care, Maternal and neonatal outcomes, Risk stratification

## Abstract

**Background:**

Cardiac disease in pregnancy is linked to significant maternal and fetal risks. A cardiac registry provides a way to systematically collect data on pregnant women with cardiac disease, enabling analysis of outcomes, care approaches, and the development of improved clinical protocols.

**Aim:**

To assess the role of a Pregnancy Heart Team (PHT) in managing cardiac disease during pregnancy, using data from a newly established registry at the National Maternity Hospital (NMH).

**Methods:**

This was a prospective study analysing registry data from women attending the joint obstetric-cardiology clinic at NMH between January 2021 and September 2024. The registry included pregnant and pre-pregnancy patients. Demographics, cardiac diagnoses, risk classification, and maternal and neonatal outcomes were collected.

**Results:**

A total of 111 women with cardiac disease were captured, encompassing 153 pregnancies. Most women (87.6%, *n* = 134) had pre-existing cardiac disease, while 12.4% (*n* = 19) were newly diagnosed during pregnancy. Rhythm disorders were the most common diagnosis (60.3%, *n* = 91). 12.4% (*n* = 19) of women received pre-conceptual counselling. Risk stratification using the modified World Health Organization (mWHO) classification showed that 33.3% (*n* = 44) were class I, 34.1% (*n* = 45) class II, 25% (*n* = 33) class II–III, and 7.6% (*n* = 10) class III; no women were classified as mWHO IV. Cardiac events occurred in 16.4% (*n* = 26), most frequently in the third trimester (42.3%, *n* = 11), highlighting this as a high-risk period.

**Conclusions:**

Registry findings highlights the importance of early, structured PHT care, to ensure optimal maternal and fetal outcomes.

## Introduction

Cardiac disease in pregnancy presents significant challenges for both maternal and fetal health, complicating an estimated 1–4% of pregnancies and contributing significantly to maternal morbidity and mortality globally [1]. Data from the European Society of Cardiology (ESC) Registry of Pregnancy and Cardiac Disease (ROPAC) show that maternal heart disease accounts for up to 15% of maternal deaths, making it the leading non-obstetric cause of maternal morbidity in high-income countries [1, 2].

The burden of cardiovascular disease in pregnancy is rising, influenced by increasing maternal age, greater use of assisted reproductive techniques, often resulting in multiple pregnancies, and the changing cardiovascular health profile of women of reproductive age [3]. With advances in modern cardiac care, many women with congenital or acquired cardiac disease now survive to adulthood and pursue pregnancy, leading to increasing numbers of high-risk pregnancies [1–4].

Physiological changes during pregnancy, including increased blood volume, elevated cardiac output, higher oxygen demand, and a prothrombotic state, may exacerbate underlying cardiac conditions [5–10]. Diagnosing these conditions can be challenging, as symptoms often overlap with those of normal pregnancy. These adaptations can result in high risk pregnancies, underscoring the importance of structured care to ensure favourable outcomes for both mother and baby.

According to the 2025 ESC Guidelines for the Management of Cardiovascular Disease in Pregnancy, the care of pregnant women with cardiac disease requires an individualised, multidisciplinary approach coordinated through a dedicated Pregnancy Heart Team (PHT) [11]. The PHT includes cardiologists, obstetricians, anaesthesiologists, neonatologists, midwives, genetic counsellors, and mental health professionals. It is recommended that these healthcare professionals are involved from the pre-conception stage through postpartum follow-up, particularly for women in mWHO class II–III or higher [11]. Early referral, structured care planning and shared decision-making are now considered a central component to optimising care, respecting maternal choice, and tailoring management plans [11].

To support evidence-based practice, registries remain crucial for collecting maternal and fetal outcome data. The ROPAC registry, established by the ESC, was the first large-scale international project to address this gap, providing critical insights into outcomes across different cardiac pathologies in pregnancy and introducing the PHT model into international guidelines [1–4, 10, 11].

This study aims to use data from a newly established cardiac registry at the National Maternity Hospital to assess the impact of the MDT, recently defined as the Pregnancy Heart Team (PHT) in the 2025 ESC guidelines, on the management of cardiac disease during pregnancy and to examine how early PHT involvement influences maternal and neonatal outcomes.

## Methods

This prospective observational study used data from The Cardiac Maternity Registry of Ireland, recently established in NMH to evaluate the effectiveness of a PHT approach in managing cardiac disease during pregnancy. It included women, both pregnant and pre-conception, who attended the joint obstetric/cardiology clinic between January 2021 and September 2024. Inclusion required either a pre-existing cardiac condition or a diagnosis of cardiac disease during pregnancy.

Data were collected prospectively from electronic patient records, including baseline cardiac and obstetric characteristics, medications, prior interventions, comorbidities, and risk factors. Maternal cardiovascular risk was classified according to the modified World Health Organization (mWHO) pregnancy risk categories. Cardiac events, maternal and fetal complications, and neonatal outcomes were recorded, with follow-up to 42 days postpartum.

Descriptive statistics summarised patient characteristics and outcomes. Categorical variables were presented as frequencies and percentages. Continuous variables were assessed for normality using the Shapiro-Wilk test and by visual inspection of histograms. Normally distributed variables were expressed as mean ± standard deviation, while non-normally distributed variables were expressed as median (interquartile range).

Analyses examined the timing and frequency of cardiac events and associations between MDT involvement and maternal and neonatal outcomes. Ethical approval was obtained from the NMH Ethics Committee. All data were anonymised prior to analysis.

**Table 1:** Patient demographics

Maternal age (mean ± SD)	34 ± 4.85 years
BMI (median, IQR)	24.86 kg/m^2^ (8.06)
NYHA class pre-pregnancy	I: 91.5% (140/153)
	II: 8.5% (13/153)
	III: 0% (0/153)
	IV: 0% (0/153)
LVEF pre-pregnancy	>55%: 93.9% (123/131)
	40–55%: 4.6% (6/131)
	<40%: 1.5% (2/131)
Maternal WHO classification	WHO I: 33.3% (44/132)
	WHO II: 34.1% (45/132)
	WHO II-III: 25% (33/132)
	WHO III: 7.6% (10/132)
	WHO IV: 0% (0/132)
Risk factors for cardiac disease	Hypertension: 9.8% (15/153)
	Dyslipidaemia: 7.8% (12/153)
	Diabetes: 0% (0/153)
	Never smoker: 84.3% (129/153)
	Ex-smoker: 9.2% (14/153)
	Current smoker: 6.5% (10/153)
Prior cardiac events	Heart failure: 12.9% (8/65)
	Cardiac arrhythmia: 64.5% (40/65)
	Ventricular arrhythmia: 8.1% (5/65)
	Thromboembolic: 3.2% (2/65)
	Endocarditis: 3.2% (2/65)
	Aortic: 0% (0/65)
	Ischaemic: 1.6% (1/65)
	Valvular: 1.6% (1/65)
	Thromboembolic (including CVA): 6.5% (4/65)
	Other: 4.8% (3/65)
Previous cardiac surgery	3.7% (21/153)
Cardiac medications pre-pregnancy	Beta blockers: 91.8% (56/63)
	Calcium channel blockers: 1.6% (1/63)
	Antiarrhythmic agents: 1.6% (1/63)
	ACEi^*^/ARB^**^: 1.6% (1/63)
	ARNi^***^: 0% (0/63)
	Spironolactone: 0% (0/63)
	Antiplatelet: 9.8% (6/63)
	Anticoagulant: 3.3% (2/63)
	Diuretic: 0% (0/63)
	Statin: 4.9% (3/63)
Major non-cardiac disease	Endocrine disorder: 23.7% (18/76)
	Haematological disorder: 11.8% (9/76)
	Inflammatory disorder: 6.6% (5/76)
	Neurological disorder: 9.2% (6/76)
	Renal disease: 1.3% (1/76)
	Mental health difficulties: 25% (19/76)
	Psychiatric disorders: 1.3% (1/76)
	Gastrointestinal disorders: 5.3% (4/76)
	Inflammatory rheumatological: 3.9% (3/76)
	Other: 39.5% (30/76)

n reflects women with available data for each variable. Not all women had baseline echocardiograms or complete pre-pregnancy risk stratification documented. *ACEi Angiotensin-converting enzyme inhibitors, **ARB: Angiotensin receptor blockers, ***ARNi: Angiotensin Receptor-Neprilysin Inhibitor

## Results

A total of 111 women, accounting for 153 pregnancies, attended the combined obstetric/cardiology clinic. Of the 153 pregnancies, complete cardiac and obstetric follow-up data were available for 134. The remaining 19 pregnancies did not contribute to follow-up analyses because they ended in early miscarriage, were preconceptual consultations only, or had incomplete follow-up. This accounts for the variation in denominators across subsequent analyses.

**Table 2:** Cardiac diagnosis & complications

Cardiac diagnosis known before pregnancy	87.6% (134/153)
Nature of cardiac disease	Rhythm: 59.4% (91/153)
	Congenital: 19.6% (30/153)
	Valvular: 16.9% (26/153)
	Cardiomyopathy/HF: 17.6% (27/153)
	Aortic: 6.5% (10/153)
	Coronary: 0.65% (1/153)
Gestation at first cardiac review (mean ± SD)	21.07 ± 8.92
Cardiac events in pregnancy & PP period	1st trimester: 23.1% (6/26)
	2nd trimester: 19.2% (5/26)
	3rd trimester: 42.3% (11/26)
	<7 days postpartum: 0% (0/26)
	7-30 days postpartum: 0% (0/26)
	Up to 42 days postpartum: 15.3% (4/26)
Nature of cardiac events in pregnancy & PP period	Heart failure: 19.2% (5/26)
	Thromboembolic: 0% (0/26)
	Haemorrhagic: 0% (0/26)
	Endocarditis: 0% (0/26)
	Aortic dissection: 0% (0/26)
	Arrhythmia: 76.9% (20/26)
	Ischaemic event: 0% (0/26)
	Valvular: 3.8% (1/26)
	Emergency cardiac surgery: 0% (0/26)
	Death: 0% (0/26)
Change in LV function	2.9% (4/134)

n reflects women with available data for each variable. Cardiac events include all pregnancies, including those that ended in miscarriage, if data were available

The mean maternal age was 34 years, with the majority having a good functional baseline, 91.5% were NYHA class I pre-pregnancy, and 93.9% had LVEF > 55% (Table 1). Most pregnancies (87.6%, *n* = 134/153) involved women with pre-existing cardiac disease, with 12.4% (*n* = 19) diagnosed during pregnancy. Rhythm disorders were the most common cardiac diagnosis, representing 59.4% (*n* = 91/153) of cases (Table 2).

Cardiac events occurred in 19.4% (*n* = 26/134) of pregnancies, most frequently in the third trimester (42.3%, *n* = 11/26), with arrhythmias accounting for 76.9% (*n* = 20/26) of events. Atrial arrhythmias were the most common (*n* = 9), followed by symptomatic premature ventricular contractions (*n* = 5), sinus tachycardia requiring admission (*n* = 3), and ventricular arrhythmias (*n* = 3), which included one episode each of non-sustained and polymorphic ventricular tachycardia (Table 2). Changes in left ventricular function were observed in 2.9% of women (*n* = 4/134), with no changes in NYHA class reported.

**Table 3:** Pregnancy outcomes

Onset of Labour	Spontaneous: 26.1% (35/134)
	Induction: 50.7% (68/134)
	Pre-labour CS: 23.1% (31/134)
Mode of delivery	Vaginal delivery: 61.1% (82/134)
	Pre-labour CS: 23.1% (31/134)
	Emergency CS: 15.6% (21/134)
Fetal concerns	Concern with fetal heart rate: 30% (2/10)
	Oligohydramnios: 10% (1/10)
	Intrauterine death: 10% (1/10)
	IUGR/SGA <5th centile: 10% (1/10)
	Preterm labour: 50% (5/10)
Maternal concerns	Gestational diabetes: 25.6% (10/39)
	Pregnancy induced hypertension: 17.9% (7/39)
	Hyperemesis gravidarum: 17.9% (7/39)
	Pre-eclampsia: 10.2% (4/39)
	Antepartum haemorrhage: 7.6% (3/39)
	Anaemia: 5.1% (2/39)
	Group B streptococcus: 5.1% (2/39)
	Placenta praevia: 2.5% (1/39)
	Maternal bacteremia: 2.5% (1/39)
	Other: 5.1% (2/39)
Pregnancy loss	Elective termination: 0% (0/8)
	Miscarriage <13 weeks: 62.5% (5/8)
	Miscarriage 13-24 weeks: 37.5% (3/8)
	Miscarriage >24 weeks: 0% (0/8)
	Ectopic: 0% (0/8)
Postpartum haemorrhage (>500ml)	20.8% (28/134)
Critical care admission duringpregnancy	CCU: 100% (2/2)
	HDU: 0% (0/2)
	ICU: 0% (0/2)
Critical care admission post-partum	9.7% (13/134)
	CCU: 15.3% (2/13)
	HDU: 76.9% (10/13)
	ICU: 7.6% (1/13)

24.8% (*n* = 38/153) were primiparous and 75.1% (*n* = 115/153) were multiparous. Regarding labour and birth, induction of labour occurred in 50.7% of cases (*n* = 68/134), with vaginal birth being the most frequent mode of delivery (61.1%, *n* = 82/134). Pre-labour caesarean sections were performed in 23.1% (*n* = 31/134), of which 26.6% (*n* = 8) were for cardiac indications, 48.3% (*n* = 15) for obstetric reasons, and 25.5% (*n* = 8) for other factors including maternal choice (Table 3).

Pregnancy complications occurred in 36.5% of cases (*n* = 49/134), with maternal complications predominating (79.5%, *n* = 39/49) and fetal complications accounting for 20.4% (*n* = 10/49). There were no maternal deaths, and 97.6% of infants were born alive (Tables 3 and 4).

Critical care admission was required in 1.5% of women during pregnancy (*n* = 2/134) and 9.7% post-partum (*n* = 13/134), primarily to high-dependency units (Table 3). Neonatal outcomes were generally favourable, with NICU admission in 5.9% (*n* = 8/134), congenital abnormalities in 1.4% (*n* = 2/134). The median gestation at time of delivery was 39 + 3 and there were very few preterm births (< 37 weeks: 2.2%, *n* = 3; <34 weeks: 1.4%, *n* = 2) (Table 4).

**Table 4:** Neonatal outcomes

NICU admission	5.9% (8/134)
Baby outcome	Alive: 97.7% (131/134)
	Stillbirth: 0.7% (1/134)
	Early neonatal death (within 7 days): 0% (0/134)
	Late neonatal death (7-28 days): 1.4% (2/134)
Birthweight	
Mean ± SD	3260.5 g ± 705.02
<2.5 kg	<2.5kg: 2.9% (4/134)
Baby congenital abnormality	1.4% (2/134)
Gestational age at delivery (median, range)	39+3 (10+3 – 42+0)

## Discussion

The findings from this study reinforce the critical role of a structured, team-based approach in managing cardiac disease in pregnancy, now formally recognised in the 2025 ESC Guidelines as the Pregnancy Heart Team (PHT) model [11]. The NMH Cardiac Registry highlights how coordinated care delivered by cardiologists, obstetricians, anaesthetists, midwives, neonatologists, and allied professionals can optimise both maternal and neonatal outcomes. The absence of maternal deaths and the high rate of neonatal survival (97.7%) in our cohort demonstrates the effectiveness of such comprehensive care.

The guidelines stress that women with mWHO class II–III and above should be managed in specialist centres by a dedicated PHT from the pre-conception stage through to postpartum follow-up [11]. Our findings support this recommendation, as structured team involvement enabled comprehensive assessments, timely interventions, and individualised care planning, which likely contributed to the observed low complication rates. Importantly, the 2025 update also emphasises shared decision-making and acknowledgement of maternal autonomy in cases where pregnancy carries substantial risk, shifting from older “absolute contraindication” messaging to transparent counselling and patient-centred decision-making [11].

A critical aspect of the PHT is early and accurate risk stratification, allowing for informed decisions about the level of care a woman will require throughout her pregnancy. The Modified World Health Organization (mWHO) Pregnancy Risk Classification provides a framework for assessing maternal risk and determining the level of surveillance they require throughout pregnancy. It is currently one of the most accurate system of risk assessment [1, 2]. Data from this study revealed that the majority of women fell into lower-risk categories with 67.4% being either mWHO I or II. Women with mWHO IV disease, where pregnancy remains extremely high-risk, benefited from tailored PHT counselling on decisions related to pregnancy continuation, mode of delivery, and postnatal follow-up.

Alongside the mWHO Pregnancy Risk Classification, the Cardiac Disease in Pregnancy II risk score (CARPREG II) score plays an essential role in refining risk stratification and decision-making for pregnant women with cardiac disease. The score categorises women into low, moderate, or high-risk groups and is frequently applied alongside the mWHO classification to support risk assessment in more complex cases. This further aids in personalising care, ensuring that each patient receives the most appropriate management for her specific risk profile.

Timing of PHT involvement is crucial. While our study shows positive outcomes with post-conception referral, guideline recommendations emphasise that pre-conception counselling provides the greatest benefit [11]. Early involvement allows for optimisation of cardiac status, accurate risk assessment, and preventative planning before the physiological stresses of pregnancy manifest. However, due to the prevalence of unplanned pregnancies and the disparities in access to preconceptual care, the first interaction between a patient and the PHT may be in the setting of an ongoing pregnancy [11]. This underscores the need for broader awareness among primary care providers and improved referral pathways to specialist PHT services.

Cardiac events occurred in 19.3% of pregnancies, with arrhythmias accounting for 76.9%. This acknowledges the guideline recognition of arrhythmias as the most common complication during pregnancy [11]. The finding that most cardiac events occurred in the third trimester or postpartum period underscores the need for specialised cardiology input during these stages, which the 2025 ESC Guidelines identify as the highest-risk periods for maternal decompensation [11].

Delivery planning is another key responsibility of the PHT. In this cohort, 23.1% (*n* = 31/134) of deliveries were planned caesarean sections, with the majority being indicated for obstetric reasons 38.7% (*n* = 12/31). This aligns with guideline recommendations, which states that vaginal delivery are generally preferred, reserving caesarean section for obstetric or select cardiovascular indications [11]. The registry also reflects guideline emphasis on planned delivery, with coordinated involvement of anaesthesia and neonatology to minimise peripartum risk.

Beyond delivery, the postpartum period remains a high-risk time for women with cardiovascular disease, particularly in the first week, due to hemodynamic shifts [8–10]. While no maternal deaths occurred in our study, this likely reflects vigilant monitoring and structured discharge planning by the PHT. Additionally, it is important to note that our cohort was not a high-risk group overall, most patients were classified as mWHO I or II and demonstrated good pre-pregnancy functional status (NYHA I), which may have also contributed to the favourable outcomes observed. The guidelines now highlight the need for extended postpartum follow-up and surveillance of women with a history of adverse pregnancy outcomes, given the high likelihood of developing cardiovascular disease later in life [11].

While this study has effectively utilised a structured registry to gather prospective data, expanding the dataset to include long-term follow-up would offer valuable insights into postpartum and inter-pregnancy outcomes. Women were followed up to 42 days postpartum, however, a limitation of our registry is that many women attending the joint obstetric/cardiology service are also seen by their primary cardiologist both pre-conception and postpartum. As a result, data from these time periods are not captured in our registry. To address this gap, the registry aims to expand to include general hospitals, ensuring comprehensive data collection for this patient cohort.

Beyond physical outcomes, the 2025 guidelines call attention to psychological health and genetic risk assessment. Cardiac disease in pregnancy poses emotional stressors, yet few studies, including ours, have systematically assessed mental health or quality of life in this population. Incorporating psychologists and genetic counsellors into the PHT, alongside education and lifestyle counselling, will be critical steps toward patient-centred care [11].

Finally, the NMH Cardiac Registry highlights the importance of registries as valuable sources of information. Just as ROPAC shaped international guidance, institutional and national registries can refine management strategies, assess long-term outcomes, and guide policy development. Expanding our registry to capture pre-conception, postpartum, long-term cardiovascular outcomes, as well as psychological impacts will align with ESC priorities and support continuous improvement.

## Conclusion

Our findings validate the Pregnancy Heart Team model outlined in the 2025 ESC Guidelines. Early referral, structured risk stratification, shared decision-making, coordinated delivery planning, and vigilant postpartum care were all shown to improve outcomes. The next step will be expanding the registry to capture genetic, psychological, and long-term cardiovascular data to ensure that care for women with cardiac disease in pregnancy continues to evolve in step with international best practice.
